# Supplementary material for: Development and evaluation of the Parenting to Reduce Child Anxiety and Depression Scale (PaRCADS): assessment of parental concordance with guidelines for the prevention of child anxiety and depression
Source: PeerJ. 2019 May 30;7:e6865. doi: 10.7717/peerj.6865 (PMC6545230; doi:10.7717/peerj.6865)
Supplement: File S1 — Original scale items in the Parenting to Reduce Child Anxiety and Depression Scale (PaRCADS). [file peerj-07-6865-s001.pdf]

**Supplemental File 1:**
**PaRCADS Items**

*Note.* This file contains the original 83 items in the scale. Items 1.8, 3.3, 5.6 and 10.6 were removed in the revised version. In the online administration of the scale, questions were personalised with a target child's name and gender, as indicated by parentheses in the items. **FP** indicates a false positive item. Scoring information is available upon on request from the authors.

*****************************

## The Parenting to Reduce Child Anxiety and Depression Scale (PaRCADS)

This survey is about your general approach to parenting [Child name]. Please answer the questions as honestly as possible.

### 1. Relationship with [Child name]

| **Please indicate how often you do the following:** | | | | | |
|---|---|---|---|---|---|
| 1.1. I let [child name] know that I love [him/her]. | **Almost Never** | **Rarely** | **Sometimes** | **Often** | **Almost Always** |
| 1.2. I show [child name] physical affection (e.g. hugs, kisses, massages, tickles). | **Almost Never** | **Rarely** | **Sometimes** | **Often** | **Almost Always** |
| 1.3. I provide encouragement to [child name] to help [him/her] feel good about [himself/herself]. | **Almost Never** | **Rarely** | **Sometimes** | **Often** | **Almost Always** |
| 1.4. I accept [child name] for who [he/she] is. | **Almost Never** | **Rarely** | **Sometimes** | **Often** | **Almost Always** |
| 1.5. I make time to talk to [child name]. | **Almost Never** | **Rarely** | **Sometimes** | **Often** | **Almost Always** |
| 1.6. I have difficulty giving [child name] my full attention when [he/she] wants to talk to me. ***(FP)*** | **Almost Never** | **Rarely** | **Sometimes** | **Often** | **Almost Always** |
| 1.7. I spend quality, one-on-one time with [child name]. | **Almost Never** | **Rarely** | **Sometimes** | **Often** | **Almost Always** |
| 1.8. I show my impatience with [child name] when [he/she] is talking to me. ***(FP)*** | **Almost Never** | **Rarely** | **Sometimes** | **Often** | **Almost Always** |

### 2. Involvement in [Child name]'s life

| **Please indicate how often you do the following:** | | | | | |
|---|---|---|---|---|---|
| 2.1. I do activities together with [child name] that [he/she] finds fun. | **Almost Never** | **Rarely** | **Sometimes** | **Often** | **Almost Always** |
| 2.2. I take time to get to know [child name]'s friends. | **Almost Never** | **Rarely** | **Sometimes** | **Often** | **Almost Always** |
| 2.3. I show interest in what [child name] is doing at school. | **Almost Never** | **Rarely** | **Sometimes** | **Often** | **Almost Always** |
| 2.4. I monitor [child name]'s performance at school (e.g. check on homework, ask teachers about how [he/she] is doing). | **Almost Never** | **Rarely** | **Sometimes** | **Often** | **Almost Always** |
| 2.5. I take steps to limit [child name]'s exposure to age-inappropriate material or any bullying on the internet. | **Almost Never** | **Rarely** | **Sometimes** | **Often** | **Almost Always** |

| | | | | | |
|---|---|---|---|---|---|
| 2.6. I give [child name] opportunities to do things that [he/she] is good at, so as to build [his/her] confidence. | **Almost Never** | **Rarely** | **Sometimes** | **Often** | **Almost Always** |
| 2.7. I discourage [child name] from trying new things that might make [him/her] feel stressed. ***(FP)*** | **Almost Never** | **Rarely** | **Sometimes** | **Often** | **Almost Always** |
| 2.8. I encourage [child name] to be responsible for some tasks in [his/her] daily life. | **Almost Never** | **Rarely** | **Sometimes** | **Often** | **Almost Always** |
| 2.9. I give [child name] the opportunity to lead the way in some activities. | **Almost Never** | **Rarely** | **Sometimes** | **Often** | **Almost Always** |
| 2.10. When [child name] is facing a problem, I try to solve it for [him/her]. ***(FP)*** | **Almost Never** | **Rarely** | **Sometimes** | **Often** | **Almost Always** |

**3. [Child name]'s relationships with others**

| **Please indicate how often you do the following:** | | | | | |
|---|---|---|---|---|---|
| 3.1. [child name] sees me building strong positive relationships with other people. | **Almost Never** | **Rarely** | **Sometimes** | **Often** | **Almost Always** |
| 3.2. I encourage [child name] to spend time with supportive adults outside the family. | **Almost Never** | **Rarely** | **Sometimes** | **Often** | **Almost Always** |
| 3.3. I encourage [child name] to spend time with the family rather than [his/her] friends on weekends. ***(FP)*** | **Almost Never** | **Rarely** | **Sometimes** | **Often** | **Almost Always** |
| 3.4 I help [child name] develop good social skills. | **Almost Never** | **Rarely** | **Sometimes** | **Often** | **Almost Always** |
| **I provide opportunities for [child name] to:** | | | | | |
| 3.5. …spend time with supportive extended family members and close family friends. | **Almost Never** | **Rarely** | **Sometimes** | **Often** | **Almost Always** |
| 3.6. …spend time with [his/her] own friends. | **Almost Never** | **Rarely** | **Sometimes** | **Often** | **Almost Always** |
| 3.7. …interact with other people in a safe environment, such as a park, or a community club or an organisation. | **Almost Never** | **Rarely** | **Sometimes** | **Often** | **Almost Always** |

**4. Rules and consequences**

Now we are going to ask you some questions about rules for [child name].
*Note:* By **rules** we mean any type of established expectations, limits or guidelines for [child]'s behaviour that [he/she] is aware of.

| **Please indicate to what extent you do the following:** | | | | | |
|---|---|---|---|---|---|
| 4.1. I have set specific, defined rules for [child name]'s behaviour. | **Yes, for almost everything** | **Yes, for many things** | **Yes, for some things** | **Hardly, only for a few things** | **No, not at all** |
| 4.2. I have set specific, defined consequences for times when [he/she] does not follow the rules. | **Yes, for almost everything** | **Yes, for many things** | **Yes, for some things** | **Hardly, only for a few things** | **No, not at all** |
| 4.3. [Child name] was involved in developing the rules. | **Yes, for almost everything** | **Yes, for many things** | **Yes, for some things** | **Hardly, only for a few things** | **No, not at all** |

| | | | | | |
|---|---|---|---|---|---|
| 4.4. I have talked with [him/her] about the reasons behind the rules. | **Yes, for almost everything** | **Yes, for many things** | **Yes, for some things** | **Hardly, only for a few things** | **No, not at all** |
| 4.5. I review or adjust the rules to adapt to [child name]'s maturity and responsibility. | **Yes, for almost everything** | **Yes, for many things** | **Yes, for some things** | **Hardly, only for a few things** | **No, not at all** |

| **Please indicate how often you do the following:** | | | | | | |
|---|---|---|---|---|---|---|
| 4.6. If [child name] gets upset with me for enforcing consequences, I let [him/her] get away with breaking the rule to keep peace. ***(FP)*** | **Almost Never** | **Rarely** | **Sometimes** | **Often** | **Almost Always** | |
| 4.7. My partner and I present different messages and expectations to [child name] regarding rules and discipline. ***(FP)*** | **Almost Never** | **Rarely** | **Sometimes** | **Often** | **Almost Always** | Not applicable, I don't have a partner. |
| 4.8. I try to understand [child name]'s perspective when [he/she] feels that a rule or consequence is unfair. | **Almost Never** | **Rarely** | **Sometimes** | **Often** | **Almost Always** | |
| 4.9. I set a good example for [child name] by following rules and laws myself. | **Almost Never** | **Rarely** | **Sometimes** | **Often** | **Almost Always** | |

## 5. Health habits

| **Please indicate how often you do the following:** | | | | | |
|---|---|---|---|---|---|
| 5.1. I encourage [child name] to eat a healthy, balanced diet, including plenty of fresh vegetables and water. | **Almost Never** | **Rarely** | **Sometimes** | **Often** | **Almost Always** |
| 5.2. In my home, treats (e.g. chips, biscuits, chocolates, or soft drinks, etc.) are readily accessible to [child name]. ***(FP)*** | **Almost Never** | **Rarely** | **Sometimes** | **Often** | **Almost Always** |
| 5.3. I practise good health habits (i.e. healthy diet, regular exercise, good sleep habits). | **Almost Never** | **Rarely** | **Sometimes** | **Often** | **Almost Always** |
| 5.4. I encourage [child name] to sleep and wake up at roughly the same time each day (even on weekends). | **Almost Never** | **Rarely** | **Sometimes** | **Often** | **Almost Always** |
| 5.5. I allow [child name] to watch TV or use an electronic device in bed before going to sleep. ***(FP)*** | **Almost Never** | **Rarely** | **Sometimes** | **Often** | **Almost Always** |
| 5.6. I encourage [child name] to nap during the day when [he/she] is tired. ***(FP)*** | **Almost Never** | **Rarely** | **Sometimes** | **Often** | **Almost Always** |

| | | | | | |
|---|---|---|---|---|---|
| 5.7. I help [child name] to engage in physical activities (e.g. by transporting [him/her] to sports or dance classes, riding a bike or walking with [him/her], encouraging [him/her] to participate in sports at school, etc.). | **Almost Never** | **Rarely** | **Sometimes** | **Often** | **Almost Always** |
| 5.8. I limit the amount of time [child name] spends watching TV or using electronic devices (e.g. the computer, mobile phones and game consoles) at home. | **Almost Never** | **Rarely** | **Sometimes** | **Often** | **Almost Always** |

**6. Home environment**

| **Please indicate how often you do the following**: | | | | | | |
|---|---|---|---|---|---|---|
| 6.1. When I have a disagreement with [child name], I discuss the issue and possible solutions with [him/her]. | **Almost Never** | **Rarely** | **Sometimes** | **Often** | **Almost Always** | |
| 6.2. When [child name] misbehaves, I point out [his/her] negative personal qualities (e.g. lazy, spoilt, selfish, etc). ***(FP)*** | **Almost Never** | **Rarely** | **Sometimes** | **Often** | **Almost Always** | |
| 6.3. I encourage [child name] to judge [his/her] actions as 'good' or 'bad', rather than judge [himself/herself] as a 'good' or 'bad' person. | **Almost Never** | **Rarely** | **Sometimes** | **Often** | **Almost Always** | |
| 6.4. When there is a conflict between family members, I encourage everyone to just brush it off and forget about it. ***(FP)*** | **Almost Never** | **Rarely** | **Sometimes** | **Often** | **Almost Always** | |
| 6.5. If I feel angry with others at home, I try to resolve the issue after I have calmed down. | **Almost Never** | **Rarely** | **Sometimes** | **Often** | **Almost Always** | |
| 6.6. I help [child name] learn ways to manage conflict, such as through staying calm and listening to others' concerns. | **Almost Never** | **Rarely** | **Sometimes** | **Often** | **Almost Always** | |
| 6.7. I discourage family members from putting down or teasing one another. | **Almost Never** | **Rarely** | **Sometimes** | **Often** | **Almost Always** | |
| 6.8. I smack [child name] when I'm angry. ***(FP)*** | **Almost Never** | **Rarely** | **Sometimes** | **Often** | **Almost Always** | |
| 6.9 When I have an argument with my partner, I criticise him/her in a personal way in front of [child name]. ***(FP)*** | **Almost Never** | **Rarely** | **Sometimes** | **Often** | **Almost Always** | Not applicable, I don't have a partner. |
| 6.10 When I have an argument with my partner, I get [child name] to be on my side. ***(FP)*** | **Almost Never** | **Rarely** | **Sometimes** | **Often** | **Almost Always** | Not applicable, I don't have a partner. |

**7. Managing emotions**

| Please indicate how often you do the following: | | | | | |
|---|---|---|---|---|---|
| 7.1. I teach [child name] ‘feeling words’ by labelling my own and [his/her] emotions as they happen in everyday life. | **Almost Never** | **Rarely** | **Sometimes** | **Often** | **Almost Always** |
| 7.2 I encourage [child name] to maintain a sense of humour in everyday situations. | **Almost Never** | **Rarely** | **Sometimes** | **Often** | **Almost Always** |
| 7.3. I help [child name] to manage [his/her] strong emotions by encouraging [him/her] to forget about them. ***(FP)*** | **Almost Never** | **Rarely** | **Sometimes** | **Often** | **Almost Always** |
| 7.4. When [child name] is upset, I encourage [him/her] to toughen up. ***(FP)*** | **Almost Never** | **Rarely** | **Sometimes** | **Often** | **Almost Always** |
| 7.5. I monitor movies or news programs that [child name] is exposed to, to see if they could be distressing to [him/her]. | **Almost Never** | **Rarely** | **Sometimes** | **Often** | **Almost Always** |
| 7.6. When [child name] is feeling angry, I tell [him/her] to stop it. ***(FP)*** | **Almost Never** | **Rarely** | **Sometimes** | **Often** | **Almost Always** |
| 7.7 I encourage [child name] to have an accepting and realistic view of [himself/herself]. | **Almost Never** | **Rarely** | **Sometimes** | **Often** | **Almost Always** |

**8. Setting goals and dealing with problems**

| Please indicate how often you do the following: | | | | | |
|---|---|---|---|---|---|
| 8.1. I encourage [child name] to work towards realistic goals and to follow through with them. | **Almost Never** | **Rarely** | **Sometimes** | **Often** | **Almost Always** |
| 8.2. When [child name] gets stuck on a problem, I explore options with [him/her] so that [he/she] can find a way through it. | **Almost Never** | **Rarely** | **Sometimes** | **Often** | **Almost Always** |
| 8.3. When talking with [child name] about a problem [he/she] has dealt with, I praise [his/her] problem-solving efforts, rather than focusing on the outcome. | **Almost Never** | **Rarely** | **Sometimes** | **Often** | **Almost Always** |
| 8.4 I help [child name] learn from [his/her] mistakes. | **Almost Never** | **Rarely** | **Sometimes** | **Often** | **Almost Always** |
| 8.5. [Child name] sees me giving up on tasks that prove to be too difficult. ***(FP)*** | **Almost Never** | **Rarely** | **Sometimes** | **Often** | **Almost Always** |

| | | | | | |
|---|---|---|---|---|---|
| 8.6. When [child name] tries a challenging new task or activity and is not at first successful, I encourage [him/her] to move on to something else. ***(FP)*** | **Almost Never** | **Rarely** | **Sometimes** | **Often** | **Almost Always** |
| 8.7 When [child name] feels discouraged while working towards a goal, I remind [child name] of the times when [he/she] managed to overcome obstacles successfully. | **Almost Never** | **Rarely** | **Sometimes** | **Often** | **Almost Always** |
| 8.8 I encourage [child name] to approach a trusted school staff member about any problems [he/she] has at school. | **Almost Never** | **Rarely** | **Sometimes** | **Often** | **Almost Always** |

**9. Dealing with negative emotions**

The following section includes questions about sadness, anxiety and anger. Everybody experiences these emotions at times. People may describe sadness, anxiety or anger as feeling "stressed", "irritable", "nervous", "on edge", "worried" or "scared". Although these emotions may be unpleasant, they can be quite useful in helping us to avoid dangerous situations or solve everyday problems.

| **Please indicate how often you do the following:** | | | | | |
|---|---|---|---|---|---|
| 9.1. I make time to listen and talk with [child name] when [he/she] is upset. | **Almost Never** | **Rarely** | **Sometimes** | **Often** | **Almost Always** |
| 9.2. I ensure that [child name] has sufficient rest and time to do relaxing activities. | **Almost Never** | **Rarely** | **Sometimes** | **Often** | **Almost Always** |
| 9.3. I help [child name] accept that [he/she] can control some things, but not everything that happens in life. | **Almost Never** | **Rarely** | **Sometimes** | **Often** | **Almost Always** |
| 9.4. When something is bothering [child name], I tell [him/her] that it's not such a big deal. ***(FP)*** | **Almost Never** | **Rarely** | **Sometimes** | **Often** | **Almost Always** |
| 9.5. When [child name] is anxious about something, I talk with [him/her] about what might have caused these feelings. | **Almost Never** | **Rarely** | **Sometimes** | **Often** | **Almost Always** |
| 9.6. When I am struggling with problems in my own life, I turn to [child name] for support. ***(FP)*** | **Almost Never** | **Rarely** | **Sometimes** | **Often** | **Almost Always** |
| 9.7. I encourage [child name] to face situations that [he/she] is afraid of. | **Almost Never** | **Rarely** | **Sometimes** | **Often** | **Almost Always** |
| 9.8. If [child name] takes steps to manage [his/her] anxiety, I praise [him/her] for doing it. | **Almost Never** | **Rarely** | **Sometimes** | **Often** | **Almost Always** |
| 9.9. I help [child name] learn the strategies that [he/she] can use for calming [himself/herself] down. | **Almost Never** | **Rarely** | **Sometimes** | **Often** | **Almost Always** |

| | | | | | |
|---|---|---|---|---|---|
| 9.10. I step in to help [child name] at the very first sign of stress or anxiety. ***(FP)*** | **Almost Never** | **Rarely** | **Sometimes** | **Often** | **Almost Always** |

**10. Getting help when needed**

| **Please indicate how likely you would be to do the following:** | | | | | |
|---|---|---|---|---|---|
| **If I noticed a persistent change in [child name]'s mood or behaviour, I would:** | | | | | |
| 10.1. …encourage [child name] to talk to me about what's going on for [him/her]. | **Very unlikely** | **Unlikely** | **Neither likely nor unlikely** | **Likely** | **Very likely** |
| 10.2. …encourage [child name] to get over it and move on. ***(FP)*** | **Very unlikely** | **Unlikely** | **Neither likely nor unlikely** | **Likely** | **Very likely** |
| 10.3. …try to determine whether the change in mood or behaviour is caused by a temporary situation, or an ongoing problem. | **Very unlikely** | **Unlikely** | **Neither likely nor unlikely** | **Likely** | **Very likely** |
| 10.4. …support [child name] in seeking professional help. | **Very unlikely** | **Unlikely** | **Neither likely nor unlikely** | **Likely** | **Very likely** |
| **If I were experiencing problems with depression or anxiety myself, I would:** | | | | | |
| 10.5. …seek professional help. | **Very unlikely** | **Unlikely** | **Neither likely nor unlikely** | **Likely** | **Very likely** |
| 10.6. …set a good example for [child name] by dealing with the problems on my own. ***(FP)*** | **Very unlikely** | **Unlikely** | **Neither likely nor unlikely** | **Likely** | **Very likely** |
