# Supplementary material for: Development and evaluation of the Parenting to Reduce Child Anxiety and Depression Scale (PaRCADS): assessment of parental concordance with guidelines for the prevention of child anxiety and depression
Source: PeerJ. 2019 May 30;7:e6865. doi: 10.7717/peerj.6865 (PMC6545230; doi:10.7717/peerj.6865)
Supplement: File S2 — Item statistics of the original PaRCADS. [file peerj-07-6865-s002.pdf]

Supplemental File S2:
Item statistics – content validity indices and discrimination indices

Item	Item-objective congruence	I-CVI Item relevance (Subscale-CVI/Ave)	Item facility/ Difficulty index	Corrected item-total point-biserial correlation r_{pb}	B-index
1.1	1	1	.94	.19	.01
1.2	.83	1	.92	.20	.08
1.3	.83	.83	.88	.37	.13
1.4	1	.83	.88	.27	.08
1.5	1	1	.76	.38	.25
1.6 [a]	1	.67	.87	.17	.08
1.7	.83	.83	.30	.32	.42
1.8 [a,b]	.83	.83 (.87)	.28	.27	.45
2.1	1	1	.42	.27	.41
2.2	1	1	.66	.25	.26
2.3	1	1	.92	.23	.08
2.4	.67	.83	.79	.20	.16
2.5	.67	.83	.86	.24	.10
2.6	.83	.83	.83	.28	.18
2.7 [a]	.83	.67	.73	.11	.07
2.8	1	1	.83	.27	.18
2.9	1	1	.44	.40	.44
2.10 [a]	1	1 (.92)	.21	.24	.31
3.1	.83	.67	.73	.29	.24
3.2	.83	.67	.39	.26	.27
3.3 [a,b]	.33	.33	.59	.15	.06
3.4	1	.83	.78	.45	.23
3.5	1	.67	.56	.27	.37
3.6	1	1	.46	.25	.36
3.7	1	.83 (.71)	.74	.26	.28
4.1	.83	1	.56	.25	.25
4.2	1	1	.35	.32	.37
4.3	.50	.67	.12	.18	.14
4.4	1	1	.64	.40	.33
4.5	1	1	.55	.46	.42
4.6 [a]	1	1	.33	.38	.34
4.7 [a]	1	.83	.23	.26	.34
4.8	.67	.83	.53	.35	.40
4.9	1	1 (.93)	.92	.26	.09
5.1	1	.67	.59	.35	.44
5.2 [a]	1	.67	.15	.12	.32
5.3	1	.67	.34	.22	.27
5.4	1	.83	.47	.25	.24
5.5 [a]	1	1	.62	.12	.14
5.6 [a,b]	.67	.33	.43	.02	-.14
5.7	1	1	.57	.25	.24
5.8	1	1 (.77)	.36	.29	.31

Note. I-CVI = Item content validity index. S-CVI/Ave = Subscale content validity index computed by averaging I-CVIs in the subscale. *B*-index = difference between the item facility of those who scored above the cut-point for concordance and the item facility of those who scored below the cut-point.
[a] False-positive item. [b] Item removed in the revised PaRCADS.

Item	Item-objective congruence	I-CVI item relevance (S-CVI/Ave)	Item facility/ Difficulty index	Corrected item-total point-biserial correlation r_{pb}	B-index
6.1	1	1	.67	.49	.35
6.2[a]	1	1	.18	.20	.13
6.3	.67	.67	.73	.30	.29
6.4[a]	.67	.50	.13	.25	.34
6.5	1	1	.53	.28	.29
6.6	1	1	.62	.38	.30
6.7	1	.83	.94	.09	.06
6.8[a]	1	1	.80	.27	.21
6.9[a]	1	1	.53	.27	.29
6.10[a]	.83	.83 (.88)	.82	.28	.19
7.1	1	1	.52	.44	.51
7.2	.83	.67	.68	.30	.29
7.3[a]	1	1	.79	.21	.17
7.4[a]	1	1	.27	.32	.51
7.5	1	.83	.78	.15	-.03
7.6[a]	1	1	.15	.30	.48
7.7	.83	.67 (.88)	.78	.36	.23
8.1	1	1	.71	.40	.20
8.2	1	1	.76	.46	.26
8.3	1	1	.58	.41	.34
8.4	.83	.83	.74	.44	.27
8.5[a]	.83	.67	.82	.26	.19
8.6[a]	1	1	.76	.15	.20
8.7	1	1	.67	.39	.30
8.8	1	.83 (.92)	.94	.14	.01
9.1	1	1	.87	.44	.14
9.2	.83	.50	.87	.27	.14
9.3	1	1	.75	.35	.26
9.4[a]	1	1	.52	.20	.40
9.5	1	1	.75	.46	.21
9.6[a]	.83	.67	.47	.16	.14
9.7	1	1	.65	.33	.32
9.8	1	1	.82	.31	.19
9.9	1	1	.54	.43	.38
9.10[a]	1	1 (.92)	.31	.13	.09
10.1	1	1	.99	.16	.02
10.2[a]	1	.83	.88	.23	.13
10.3	1	1	.97	.08	.03
10.4	1	1	.86	.11	.15
10.5	1	.83	.83	.20	.03
10.6[a,b]	1	.67 (.89)	.45	.15	.26

Note. I-CVI = Item content validity index. S-CVI/Ave = Subscale content validity index computed by averaging I-CVIs in the subscale. *B*-index = the difference between the item facility of those who scored above the cut-point for concordance and the item facility of those who scored below the cut-point.
[a] False-positive item. [b] Item removed in the revised PaRCADS.
