# Supplementary material for: Development and evaluation of the Parenting to Reduce Child Anxiety and Depression Scale (PaRCADS): assessment of parental concordance with guidelines for the prevention of child anxiety and depression
Source: PeerJ. 2019 May 30;7:e6865. doi: 10.7717/peerj.6865 (PMC6545230; doi:10.7717/peerj.6865)
Supplement: Table S1 — Descriptive statistics for parent-reported mental health characteristics and concerns about child’s risk of developing anxiety or depression (N = 355). [file peerj-07-6865-s003.pdf]

Supplemental Table S1:
Descriptive statistics for parent-reported mental health characteristics and concerns about child's risk (*N* = 355)

	N	%
**Parent history of mental health problem**		
No	123	34.6
Yes, past only	169	47.6
Yes, current for at least 2 weeks	21	5.9
Yes, past and current	42	11.8
**Parent history of mental health diagnosis, past or current**[a]		
Depression	66	18.6
Anxiety	111	31.3
Others (e.g. attention, relationship problems)	116	32.7
None	123	34.6
**Parental concerns about child's risk of developing depression**		
Not at all	54	15.2
A little	175	49.3
Yes	73	20.6
Very much so	53	14.9
**Parental concerns about child's risk of developing anxiety**		
Not at all	41	11.5
A little	143	40.3
Yes	92	25.9
Very much so	79	22.3
**Child history of mental health or behavioural diagnosis**[a]		
Depression	2	0.6
Any anxiety disorder	31	8.7
Autism	14	3.9
Asperger's disorder	9	2.5
Attention-deficit/hyperactivity disorder	22	6.2
Intellectual disability	2	0.6
Oppositional defiant disorder	5	1.4
Conduct disorder	1	0.3
Other (e.g. learning disorder, anger)	116	32.7
Child has never been diagnosed, however I believe my child has experienced some emotional or behavioural problems	211	59.4
**Child current mental health or behavioural problem**[a]		
Depression	5	1.4
Any anxiety disorder	33	9.3
Autism	13	3.7
Asperger's disorder	9	2.5
Attention-deficit/hyperactivity disorder	24	6.8
Intellectual disability	2	0.6
Oppositional defiant disorder	4	1.1
Conduct disorder	1	0.3
Other (e.g. learning disorder, Tic disorder, anger, stress, etc.)	83	23.4
No formal diagnosis, however I believe [he/she] is experiencing some emotional or behavioural problems	36	10.1
No current mental health or behavioural problems	195	54.9

Note. Percentages are based on parents' report, regardless of their child's participation in the study.
[a]Percentages exceed 100% as multiple responses were allowed.
