# Supplementary material for: Development and evaluation of the Parenting to Reduce Child Anxiety and Depression Scale (PaRCADS): assessment of parental concordance with guidelines for the prevention of child anxiety and depression
Source: PeerJ. 2019 May 30;7:e6865. doi: 10.7717/peerj.6865 (PMC6545230; doi:10.7717/peerj.6865)
Supplement: Table S2 — Means, Standard Deviations, and t-test Results Comparing PaRCADS Total Scores for Participants Above and Below Borderline/Clinical Cut-off on the Revised Children’s Anxiety and Depression Scale (RCADS-25). [file peerj-07-6865-s004.pdf]

Supplemental Table S2:
Means, Standard Deviations, and *t*-test Results Comparing PaRCADS Total Scores for Participants Above and Below Borderline/Clinical Cut-off on the RCADS-25

	n	*M*	*SD*	*t*	*df*	*p*	Hedges *g*
**RCADS-P Anxiety**				.64*	71.10	.525	-0.08
Normal	309	50.72	10.94				
Borderline/Clinical	46	49.85	8.23				
**RCADS-P Depression**				2.81	353	.005	-0.37
Normal	280	51.42	10.61				
Borderline/Clinical	75	47.57	10.21				
**RCADS-P Total**				2.64	353	.009	-0.37
Normal	294	51.28	10.57				
Borderline/Clinical	61	47.36	10.39				
**RCADS-C Anxiety**				1.29	340	.198	-0.34
Normal	327	52.18	10.90				
Borderline/Clinical	15	48.47	10.70				
**RCADS-C Depression**				-.41*	20.90	.687	0.06
Normal	326	51.98	11.09				
Borderline/Clinical	16	52.63	5.77				
**RCADS-C Total**				-.24	340	.812	0.06
Normal	324	51.98	11.05				
Borderline/Clinical	18	52.61	7.89				

Note. *equal variances not assumed.
