# Supplementary material for: Development and evaluation of the Parenting to Reduce Child Anxiety and Depression Scale (PaRCADS): assessment of parental concordance with guidelines for the prevention of child anxiety and depression
Source: PeerJ. 2019 May 30;7:e6865. doi: 10.7717/peerj.6865 (PMC6545230; doi:10.7717/peerj.6865)
Supplement: Supplemental Information 1 [file peerj-07-6865-s005.docx]

Contents

[Participant characteristics 2](#_Toc530131340)

[AQoL 9](#_Toc530131341)

[GF 9](#_Toc530131342)

[KIDSCREEN 9](#_Toc530131343)

[RCADS 10](#_Toc530131344)

[CRPBI - Acceptance 10](#_Toc530131345)

[PCS 11](#_Toc530131346)

[PaRCADS 11](#_Toc530131347)

## Participant characteristics

| **Name** | **Label** | **Values (for categorical data)**  **None: use interval data as available** |
| --- | --- | --- |
| **id_research** | **participant research id** | **None** |
|  |  |  |
| **parent_age** | **parent age** | **None** |
|  |  |  |
| **parent_gender** | **parent gender** | 1 =male |
|  |  | 2 =female |
|  |  |  |
| **parent_education** | **parent education level** | 1 =Year 7 to Year 11 |
|  |  | 2 =Year 12 |
|  |  | 3 =Trade or apprenticeship |
|  |  | 4 =Other TAFE/technical certificate |
|  |  | 5 =Diploma |
|  |  | 6 =Advanced diploma/Associate degree |
|  |  | 7 =Bachelor degree |
|  |  | 8 = Bachelor honours/Graduate diploma/Graduate certificate |
|  |  | 9 =Masters degree/Doctoral degree |
|  |  |  |
| **parent_employment** | **parent employment** | 1 =unemployed |
|  |  | 2 =working full time |
|  |  | 3 =working part time |
|  |  |  |
|  |  |  |
| **parent_study** | **parent study status** | 1 =not studying |
|  |  | 2 =studying full time |
|  |  | 3 =studying part time |
|  |  |  |
|  |  |  |
| **language_raw** | **language_raw** | 1 =No, English only |
|  |  | 2 =Arabic |
|  |  | 3 =Cantonese |
|  |  | 4 =French |
|  |  | 5 =German |
|  |  | 6 =Greek |
|  |  | 7 =Italian |
|  |  | 8 =Mandarin |
|  |  | 9 =Maltese |
|  |  | 10 =Spanish |
|  |  | 11 =Tagalog |
|  |  | 12 =Vietnamese |
|  |  | 13 =Others |
|  |  |  |
| **english_language_only_recode** | **english language only** | 1 =English |
|  |  | 2 =Language other than English |
|  |  |  |
| **indigenous_status** | **identifies self as Aboriginal/Torres Strait Islander** | 1 =no |
|  |  | 2 =yes |
|  |  | 3 =I would prefer not to say |
|  |  |  |
|  |  |  |
| **marital_status** | **parent_relationship status** | 1 =single |
|  |  | 2 =married/defacto |
|  |  | 3 =separated or divorced |
|  |  | 4 =widowed |
|  |  |  |
| **number_of_children** | **number of children** | **None** |
|  |  |  |
| **dyad_relationship** | **dyadic relationship** | 1 =Mother |
|  |  | 2 =Father |
|  |  | 3 =Step-mother |
|  |  | 4 =Step-father |
|  |  | 5 =Grandmother |
|  |  | 6 =Grandfather |
|  |  | 7 =Guardian |
|  |  | 8 =Others |
|  |  |  |
| **living_arrangement** | **child living arrangement** | 1 =Both parents/guardians in same home |
|  |  | 2 =Both parents/guardians in separate homes |
|  |  | 3 =One parent/guardian (me) |
|  |  | 4 =One parent/guardian (other parent) |
|  |  | 5 =Other |
|  |  |  |
| **child_participated** | **child participation status** | 1 =No, child not participating with parent |
|  |  | 2 =Yes, child participating with parent |
|  |  |  |
| **child_crpbi30_administered** | **child_crpbi_administration** | 1 =No, not applicable |
|  |  | 2 =Yes |
|  |  |  |
| **child_sex** | **child_gender** | 1 =boy |
|  |  | 2 =girl |
|  |  |  |
| **child_grade** | **child grade (school year)** | 3 =Grade 3 |
|  |  | 4 =Grade 4 |
|  |  | 5 =Grade 5 |
|  |  | 6 =Grade 6 |
|  |  |  |
| **child_age** | **child age (years)** | **None** |
|  |  |  |
| **parent_depression** | **parent_depression diagnosis** | 0 =none |
|  |  | 1 =yes |
|  |  |  |
| **parent_any_anxiety** | **parent_any_anxiety diagnosis** | 0 =none |
|  |  | 1 =yes |
|  |  |  |
| **parent_other_mh_diagnosis** | **parent_other MH diagnosis** | 0 =none |
|  |  | 1 =yes |
|  |  |  |
| **parent_no_mh__diagnosis** | **parent_MH diagnosis question not applicable_i.e.no diagnosis** | 0 =no |
|  |  | 1 =yes |
|  |  |  |
| **parent_hx_mhproblem_recode** | **parent_history of mental health problem** | 1 =None |
|  |  | 2 =Yes, only in the past |
|  |  | 3 =Yes, only currently |
|  |  | 4 =Yes, past and current |
|  |  | 5 =Others without time specified |
|  |  | 6 =Others, not mental health problems |
|  |  |  |
| **parent_hx_mhdiagnosis_summative** | **parent_hx_mhdiagnosis_summative** | **None** |
|  |  |  |
| **parent_hx_mhdiagnosis_recode** | **parent_hx_mhdiagnosis_presence** | 1 =none |
|  |  | 2 =present |
|  |  |  |
| **parent_current_mhproblem** | **parent_current_mhproblem** | **None** |
|  |  |  |
| **parent_current_mhproblem_recode** | **parent_current_mhproblem_presence** | 0 =none |
|  |  | 1 =present |
|  |  |  |
| **parental_concern_depression** | **parental concern_child depression** | 1 =Not at all |
|  |  | 2 =A little |
|  |  | 3 =Yes |
|  |  | 4 =Very much so |
|  |  |  |
| **parental_concern_anxiety** | **parental concern_child anxiety** | 1 =Not at all |
|  |  | 2 =A little |
|  |  | 3 =Yes |
|  |  | 4 =Very much so |
|  |  |  |
| **child_depression** | **child_depression diagnosis** | 0 =none |
|  |  | 1 =yes |
|  |  |  |
| **child_anxiety** | **child_any anxiety diagnosis** | 0 =none |
|  |  | 1 =yes |
|  |  |  |
| **child_autism** | **child_autism diagnosis** | 0 =none |
|  |  | 1 =yes |
|  |  |  |
| **child_asperger** | **child_asperger’s disorder** | 0 =none |
|  |  | 1 =yes |
|  |  |  |
| **child_adhd** | **child_ADHD diagnosis** | 0 =none |
|  |  | 1 =yes |
|  |  |  |
| **child_intellectual** | **child_intellectual disability** | 0 =none |
|  |  | 1 =yes |
|  |  |  |
| **child_odd** | **child_oppositional defiant disorder** | 0 =none |
|  |  | 1 =yes |
|  |  |  |
| **child_cd** | **child_conduct disorder** | 0 =none |
|  |  | 1 =yes |
|  |  |  |
| **child_others** | **child_others e.g. learning, anger, etc.** | 0 =none |
|  |  | 1 =yes |
|  |  |  |
| **child_no_formal_diagnosis** | **child_no formal diagnosis but parent reported problem** | 0 =none |
|  |  | 1 =yes |
|  |  |  |
| **child_current_depression** | **child_current depression** | 0 =no |
|  |  | 1 =yes |
|  |  |  |
| **child_current_any_anxiety** | **child_current_any_anxiety** | 0 =no |
|  |  | 1 =yes |
|  |  |  |
| **child_current_autism** | **child_current autism** | 0 =no |
|  |  | 1 =yes |
|  |  |  |
| **child_current_asperger** | **child_current asperger’s disorder** | 0 =no |
|  |  | 1 =yes |
|  |  |  |
| **child_current_adhd** | **child_current ADHD diagnosis** | 0 =no |
|  |  | 1 =yes |
|  |  |  |
| **child_current_intellectual** | **child_current intellectual disability** | 0 =no |
|  |  | 1 =yes |
|  |  |  |
| **child_current_odd** | **child_current oppositional defiant disorder** | 0 =no |
|  |  | 1 =yes |
|  |  |  |
| **child_current_cd** | **child_current conduct disorder** | 0 =no |
|  |  | 1 =yes |
|  |  |  |
| **child_current_others** | **child_current others e.g. learning, anger, etc.** | 0 =no |
|  |  | 1 =yes |
|  |  |  |
| **child_current_no_formal_diagnosis** | **child_no formal diagnosis but parent reported problem** | 0 =no |
|  |  | 1 =yes |
|  |  |  |
| **child_current_mhproblem_parent_nonresponse** | **child_current mh question** | 0 =answered |
|  |  | 1=did not answer as it’s not applicable |
|  |  |  |
| **Child_hx_mhdiagnosis** | **child_hx_mhdiagnosis** | **None** |
|  |  |  |
| **Child_hx_mhdiagnosis_recode** | **child_hx_mhdiagnosis_presence** | 0 =none |
|  |  | 1 =present |
|  |  |  |
| **child_current_mh_formaldiagnosis_summative** | **child_current_mh_formaldiagnosis_summative** | **None** |
|  |  |  |
|  |  |  |
| **child_current_mh_formaldiagnosis_recode** | **child_current_mh_formaldiagnosis_presence** | 0 =none |
|  |  | 1 =present |
|  |  |  |

## AQoL

| **Name** | **Label** | **Values** |
| --- | --- | --- |
| **aqol1 to aqol35** | **aqol** | *Ordinal data – varied values across items*  Please refer to data file for specific label/meaning of each value or obtain the original questionnaire from the authors of AQoL-8D) |

## GF

| **Name** | **Label** | **Values** |
| --- | --- | --- |
| **GF_bl_q1_reversed to GF_bl_q12** | **General family functioning-Parent** | *Ordinal data*  1 =strong agree  2 =agree  3 =disagree  4 =strongly disagree |

## KIDSCREEN

| **Name** | **Label** | **Values** |
| --- | --- | --- |
| **KY27PHY1_reversed to**  **KP27SCH4** | **KIDSCREEN-Child_Physical wellbing to**  **KIDSCREEN-Parent_School environment** | *Ordinal data– varied values across dimensions*  Please refer to data file for specific label/meaning of each value or obtain the original questionnaire from the authors of the KIDSCREEN-27) |

## RCADS

| **Name** | **Label** | **Values** |
| --- | --- | --- |
| **RCADS25_C_1 to**  **RCADS25_P_25** | **RCADS-Child to**  **RCADS-Parent** | *Ordinal data*  1 =never  2 =sometimes  3 =often  4 =always |

| **Name** | **Label** | **Values** |
| --- | --- | --- |
| **ACC_C_bl_q1 to**  **ACC_C_bl_q10** | **CRPBI-Acceptance-Child** | *Ordinal data*  1 =not like  2 =somewhat like  3 =a lot like |
| **ACC_P_bl_q1 to**  **ACC_P_bl_q10** | **CRPBI-Acceptance-Parent** | *Ordinal data*  1 =not like  2 =somewhat like  3 =a lot like |

## CRPBI - Acceptance

| **Name** | **Label** | **Values** |
| --- | --- | --- |
| **PCS_C_bl_q1 to**  **PCS_C_bl_q8** | **Psychological Control Scale-Child** | *Ordinal data*  1 =not like  2 =somewhat like  3 =a lot like |
| **PCS_P_bl_q1 to**  **PCS_P_bl_q8** | **Psychological Control Scale-Parent** | *Ordinal data*  1 =not like  2 =somewhat like  3 =a lot like |

## PCS

## PaRCADS

| **Name** | **Label** | **Values** |
| --- | --- | --- |
| **PaRCADS_bl_d1_q1 to**  **PaRCADS_bl_d10_q6** | **PaRCADS_subscale1 to**  **PaRCADS_subscale10** | *Ordinal data – varied values across subscales*  Please refer to data file for specific label/meaning of each value or obtain the original questionnaire from the authors of the PaRCADS) |
