# Supplementary material for: Development and evaluation of the Parenting to Reduce Child Anxiety and Depression Scale (PaRCADS): assessment of parental concordance with guidelines for the prevention of child anxiety and depression
Source: PeerJ. 2019 May 30;7:e6865. doi: 10.7717/peerj.6865 (PMC6545230; doi:10.7717/peerj.6865)
Supplement: Supplemental Information 2 [file peerj-07-6865-s006.docx]

## Participant characteristics

| **Name** | **Label** | **Values (for categorical data)**  **None: use interval data as available** |
| --- | --- | --- |
| **Research_id** | **participant research id** | **None** |
|  |  |  |
| **Years** | **At least 5 years of experience** | 1= less than 5 years |
|  |  | 2 =more than 5 years |
|  |  |  |
| **Receipt_mode** | **Preference for receipt of review materials** | 1 =view the materials online |
|  |  | 2 =receive the materials by post |
|  |  |  |
| **Return_mode** | **Preference for return of review responses** | 1 =via this online survey |
|  |  | 2 =by email |
|  |  | 3 =by post |
|  |  |  |
| **Qualification** | **Highest level of education** | 1= less than high school degree |
|  |  | 2 = high school graduate |
|  |  | 3 = some college but no degree |
|  |  | 4 = associate degree in college |
|  |  | 5 =bachelor degree in college |
|  |  | 6 = master’s degree |
|  |  | 7 = doctoral degree |
|  |  | 8 =professional degree (JD, MD) |
|  |  |  |
| **Profession_1** | **Involved in education** | 1 =involved in education |
|  |  |  |
|  |  |  |
| **Profession_2** | **Involved in clinical or psychological treatment** | 1 =involved in clinical or psychological treatment |
|  |  |  |
| **Profession_3** | **Involved in research** | 1= Involved in research |
|  |  |  |
|  |  |  |

## Ratings

| **Name** | **Label** | **Values** |
| --- | --- | --- |
| **Ob1.1 to**  **Ob10.6** | **Item 1.1__Item-Objective rating to**  **Item10.6_Item-Objective rating** | 1 =yes |
|  |  | 2 =no |
|  |  |  |
| **Re1.1 to**  **Re10.6** | **Item1.1_Item-Relevance rating to**  **Item10.6_Item-Relevance rating** | 1 =not relevant |
|  |  | 2 =somewhat relevant |
|  |  | 3 =relevant |
|  |  | 4 =very relevant |
